# Assessment and diagnosis: a rational approach

**Published:** 2017-02-10

**Authors:** Jeremy Hoffman, Matthew Burton

**Affiliations:** 1 Academic Clinical Fellow: International Centre for Eye Health and Specialist Registrar, Moorfields Eye Hospital, London, UK.; 2 Professor: International Centre for Eye Health and Consultant Ophthalmologist: Moorfields Eye Hospital, London, UK.

The ocular surface is critical to the health of the eye and essential for good visual functioning. It is a complex, integrated system involving the cornea, conjunctiva, tear film, lacrimal gland, nasolacrimal system and the eyelids (incorporating the meibomian glands and lashes). The normal physiological function of the ocular surface depends on the interaction of these different components. Working together, they maintain a clear optical surface, keep the eye from drying out, and protect it from trauma and infection. Changes in the structure and function of any of the ocular surface components can disrupt its delicate balance and lead to pathology.

Ocular surface diseases have a relatively limited set of symptoms and signs, and a systematic approach to assessing and diagnosing these conditions is therefore necessary.

## History

Because patients with ocular surface problems present with a limited range of symptoms and signs, taking a detailed history is very important. Ask patients whether they have experienced, orare experiencing, any of the following:

- **Reduced vision** (mild blurring can occur if the tear film is disturbed; a more severe visual disturbance suggests corneal or other disease)
- **Redness**
- **Irritation or gritty sensation** (suggests epithelial disturbance)
- **Itching** (suggests allergy)
- **Pain** (sharp pain suggests a corneal problem or foreign body; a duller ache may suggest uveal or scierai inflammation)
- **Purulent discharge**
- **Watering,** whether from lacrimation (increased tear production) or epiphora (decreased tear drainage)

**Figure:**
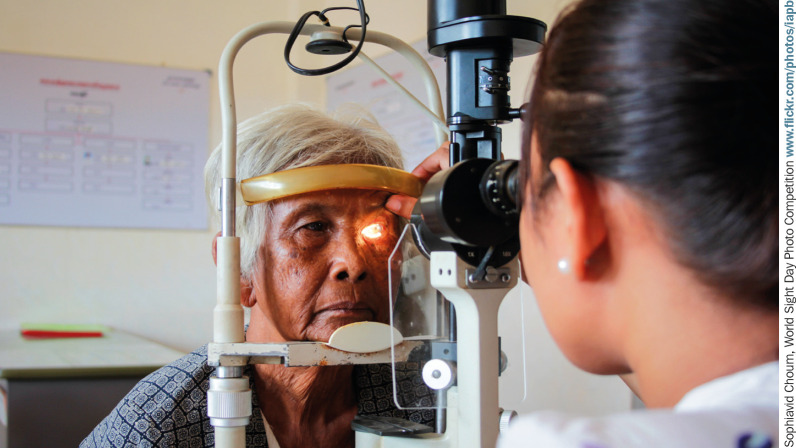
Examining the ocular surface. CAMBODIA

It is important to take a careful note of when and how the problem developed. You need to ask if there has been a history of trauma or a foreign body. In some settings, contact lens use is common and you need to ask about this. If patients do use contact lenses, ask how they clean and use them.

**ABOUT THIS ISSUE**

Many diseases can affect the ocular surface. Their frequency and severity varies from region to region, often depending on the local climate. Ocular surface diseases can affect both eyesight and quality of life, and – in severe cases – cause blindness. Because they have a limited number of symptoms and signs, and can appear very similar in presentation, patients can be misdiagnosed and hence poorly managed. In this issue, we offer a systematic approach to assessing and diagnosing common ocular surface diseases and look in detail at general management principles, including how to control inflammation. Other articles discuss ocular allergy, pterygium and squamous cell carcinoma. In the middle of the issue we also have a poster with useful information about common ocular surface conditions and their primary management.

— ***Elmien Wolvaardt Ellison (Editor)***

## Examination

Your examination of the ocular surface needs to be systematic. A stepwise approach helps to ensure that important things are not missed.

- **Vision.** Start by assessing the uncorrected, pinhole and best corrected visual acuity.
- **Eyelids.** Examine the lid position and closure and check for entropion (when the eyelid turns in on itself), trichiasis (lashes touching the eye) and lagophthalmos (a gap between the upper and lower lid when the eyes are closed). Examine the lid margin and meibomian gland openings for abnormal positions, inflammation and plugging with secretions. Try to express the meibomian glands, using gentle pressure.
- **Tears.** Assess the quality of the tear film by looking for discharge or debris and the tear meniscus height (to give an idea of quantity). Check the tear break-up time by instilling a drop of fluorescein and timing how long it takes for the tear film to disperse. A tear break-up time of less than 10 seconds is abnormal. Finally, perform Schirmer's test by placing a testing strip in the inferior conjunctival fornix and asking the patient to close their eyes for five minutes. A normal result is > 15 mm. Less than this suggests insufficient tear production, to varying degrees: mild is 9–14 mm, moderate is 4–8 mm and severe is <4 mm.
- **Bulbar conjunctiva and sciera.** Assess inflammation, scarring, haemorrhages and abnormal swellings such as pinguecula, pterygium or possible malignancies.
- **Tarsal conjunctiva.** Evert the upper and lower lids. Look for scarring, foreign body defects, inflammatory membranes, papillae and follicles.
- **Corneal epithelium.** Using a torch, look for foreign bodies, infiltrates, oedema and deposits. Is the light reflected off the eye's surface shiny (healthy), or rough and/or dull? Also test for corneal sensation, which may be reduced due to infection with herpes simplex or zoster.
- **Corneal stroma.** Look for stromal opacities. Assess the size, location, pattern and depth. Opacities may be scars or active inflammatory infiltrates. Look for blood vessels: active vessels have blood flowing, inactive have a clear, grey outline without blood.
- **Corneal endothelium.** Look for any guttata, Descemet folds and the presence and type of any deposits (blood, keratic precipitates or pigment).

## Diagnosis

Problems affecting the ocular surface broadly divide into non-infectious and infectious conditions. They present with a limited range of symptoms. The pattern of symptoms can often help to differentiate between conditions. In Table 1 we outline the typical symptom pattern for some of the commoner conditions. For example, if the person mainly complains of itching, then allergic conjunctivitis needs to be considered as a possible cause.

The symptoms of these different conditions can overlap. Therefore, a careful examination is critical to reaching an accurate diagnosis. Although not exhaustive, there is a list of common and important ocular surface conditions on pages 50–51, detailing their presenting features and some example photographs.

**Table 1:** Symptom and signs of common conditions

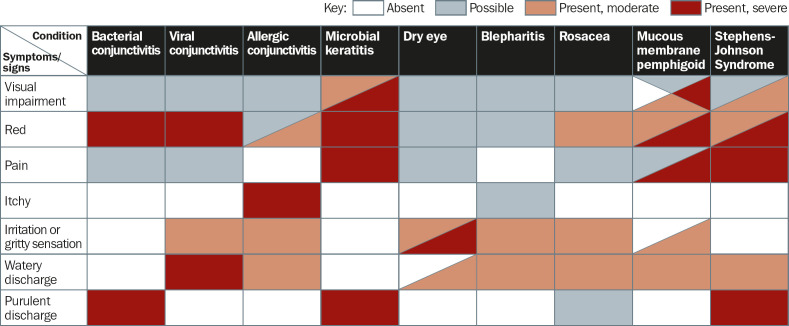

**Understanding the ocular surface**

**Jeremy Hoffman** and **Matthew Burton**

**Figure 1:**
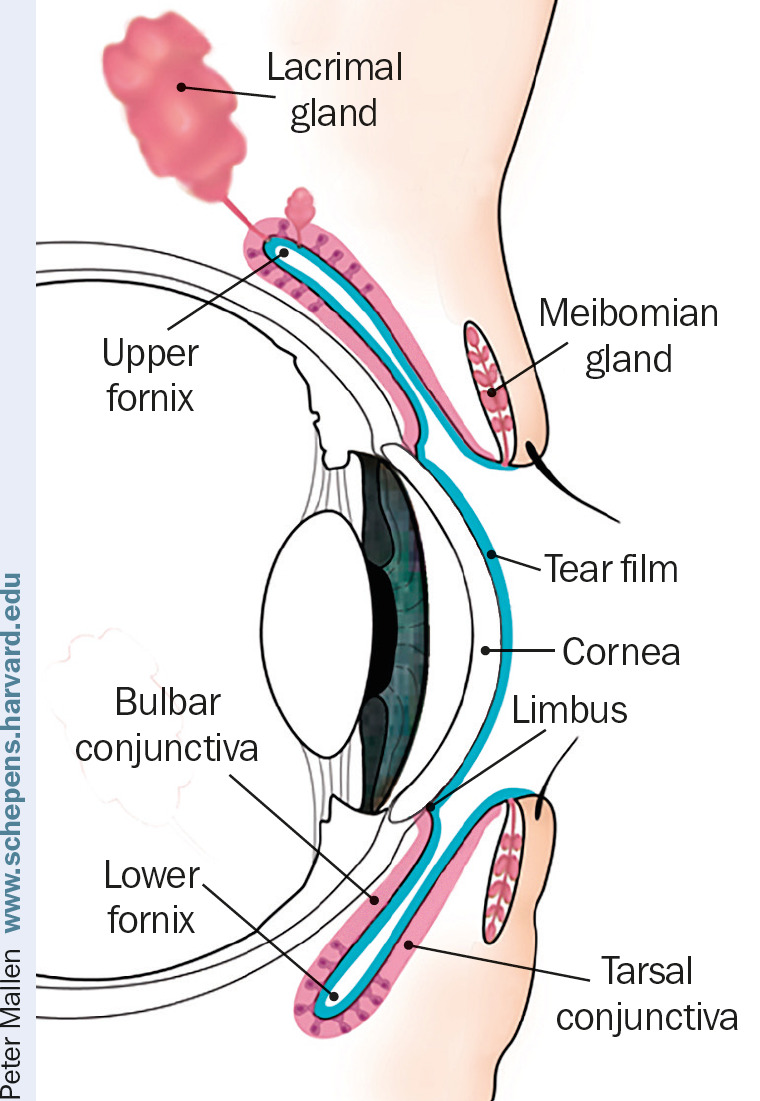
Anatomy of the upper eyelid and anterior segment of the eye in cross-section

The ocular surface consists of the cornea, conjunctiva, tear film, lacrimal gland, nasolacrimal system and the eyelids (incorporating the meibomian glands and lashes), each of which is described in detail below. Figure 1 shows the anatomy of the upper eyelid and anterior segment of the eye in cross-section.

**Cornea**

The cornea is the most powerful refracting component of the eye. Together with the lens, it focuses light on the retina. The central 4 mm zone is critical for good vision. The cornea is made up of five layers: epithelium, Bowman's layer, stroma, Descemet's membrane and endothelium. The normal cornea does not have blood vessels; it gains oxygen and nutrients through diffusion from the aqueous, from limbal blood vessels and from the atmosphere. The cornea is very sensitive; there is dense innervation by fine nerve fibres from the trigeminal nerve. Normal corneal sensation is essential for a healthy intact epithelial surface, tear function and protection through the blink reflex.

If damaged, the corneal epithelium can regenerate, so simple abrasion injuries can heal without scarring. However, if the stem cells that repopulate the corneal epithelial surface are damaged, for example by a chemical injury, the resulting epithelium is abnormal and clarity is lost. Corneal clarity also depends on there being a highly ordered arrangement of collagen fibres within the stroma. These deeper layers are unable to regenerate well and often heal with scarring. In addition, the cornea needs to be maintained in a relatively dehydrated state by the action of the endothelial cell layer. If this is not functioning well, the cornea becomes oedematous and opaque.

**Conjunctiva**

The conjunctiva is composed of an epithelial layer overlaying a loose connective tissue (stroma). It covers the eye from the edge of the cornea (limbus) to the fornices and the inside surface of the eyelids. It contains specialised goblet cells that produce the mucus layer of the tear film. In the stromal layer of the conjunctiva, there are immune system cells that defend against infection. Sometimes lymphoid cells are recruited and gather together to form visible follicles, particularly on the tarsal conjunctival surface. Papillae, which form in the tarsal conjunctiva, are dome-like swellings with inflammatory cells, oedema and a dilated blood vessel. Conjunctival scarring develops in some chronic inflammatory ocular surface conditions, with shortened fornices, symblepharon (adhesions between the eye lid and globe) and distortion of the eyelids.

**Tear film**

The tear film is made up of three layers. The outer lipid layer (produced by the meibomian glands) reduces evaporation of the middle aqueous layer (produced by the lacrimal gland), with the inner mucin layer (produced by goblet cells) helping to stabilise the aqueous layer on the corneal epithelium. A good tear film helps to maintain a well-hydrated, healthy corneal epithelium and a clear optical surface, and it protects against infection.

**Lacrimal gland**

The lacrimal gland sits in the supero-lateral region of the orbit. Fine ducts open into the upper fornix, delivering lacrimal fluid to the ocular surface. Secretion of tear fluid is controlled by the parasympathetic nervous system. Problems with the gland itself, obstruction of the ducts (by scarring) and neurological problems can all result in reduced aqueous tear production.

**Nasolacrimal system**

The nasolacrimal system drains tear fluid from the surface of the eye. Fluid is collected through the punctae and passes along the canaliculi into the lacrimal sac. From the sac, the fluid passes down the nasolacrimal duct and drains into the nasal cavity. Obstruction at any point along the system can result in a watery eye (epiphora) and predispose the eye to infection.

**Eyelids**

Eyelids protect the eyes by covering them. They are formed of several layers: skin, the orbicularis muscle, the tarsal plate (including the meibomian glands), and the conjunctiva.

